# Novel Drugs with High Efficacy against Tumor Angiogenesis

**DOI:** 10.3390/ijms23136934

**Published:** 2022-06-22

**Authors:** Shiyu Qi, Shoulong Deng, Zhengxing Lian, Kun Yu

**Affiliations:** 1 College of Animal Science and Technology, China Agricultural University, Beijing 100193, China; s20203040579@cau.edu.cn; 2 National Health Commission (NHC) of China Key Laboratory of Human Disease Comparative Medicine, Institute of Laboratory Animal Sciences, Chinese Academy of Medical Sciences and Comparative Medicine Center, Peking Union Medical College, Beijing 100021, China; dengshoulong@cnilas.org

**Keywords:** anti-tumor angiogenesis, VEGF/VEGFR, Tie/Ang, HSP inhibitors, novel drugs

## Abstract

Angiogenesis is involved in physiological and pathological processes in the body. Tumor angiogenesis is a key factor associated with tumor growth, progression, and metastasis. Therefore, there is great interest in developing antiangiogenic strategies. Hypoxia is the basic initiating factor of tumor angiogenesis, which leads to the increase of vascular endothelial growth factor (VEGF), angiopoietin (Ang), hypoxia-inducible factor (HIF-1), etc. in hypoxic cells. The pathways of VEGF and Ang are considered to be critical steps in tumor angiogenesis. A number of antiangiogenic drugs targeting VEGF/VEGFR (VEGF receptor) or ANG/Tie2, or both, are currently being used for cancer treatment, or are still in various stages of clinical development or preclinical evaluation. This article aims to review the mechanisms of angiogenesis and tumor angiogenesis and to focus on new drugs and strategies for the treatment of antiangiogenesis. However, antitumor angiogenic drugs alone may not be sufficient to eradicate tumors. The molecular chaperone heat shock protein 90 (HSP90) is considered a promising molecular target. The VEGFR system and its downstream signaling molecules depend on the function of HSP90. This article also briefly introduces the role of HSP90 in angiogenesis and some HSP90 inhibitors.

## 1. Introduction

Tumor development is primarily dependent on the vascular supply, which is facilitated by angiogenic activity within malignant tissues. The inhibition of angiogenesis is considered to be a promising therapeutic approach. Angiogenesis is controlled by the interaction of certain biomolecules produced in the body. It is a process in which new blood vessels are formed by “sprouting” from pre-existing blood vessels, and this process is involved in the physiological and pathological processes of the body [1]. The process of angiogenesis is complex and mainly includes the production of proteases, endothelial cell migration and proliferation, the formation of vascular tubes, the anastomosis of newly formed tubes, the synthesis of new basement membranes, and the incorporation of envelope and smooth muscle cells [2,3].

The biological process of angiogenesis is thought to be important for normal physiological growth, tissue regeneration and wound healing [4]. Under normal conditions, angiogenesis occurs only in processes, such as embryonic development, the female reproductive cycle, and wound repair [5]. However, abnormal angiogenesis is a key mediator and major process of cancer development. Furthermore, tumor angiogenesis is a key factor associated with tumor growth, progression, and metastasis [6]. Angiogenesis is required for the development and growth of solid tumors larger than 1–2 mm^3^ in size [7]. Solid tumors depend on neovascularization for continued growth [8].

Tumor angiogenesis is achieved through a series of sequential steps that further lead to cancer development. It is caused by an imbalance between pro- and antiangiogenic modulators in the tumor microenvironment [6]. The process of angiogenesis is mainly initiated by the tumor itself. When a malignant tumor grows to a certain size, it causes the cells to become hypoxic [9]. Hypoxia is characterized by O_2_ tension levels below 5–10 mmHg, which is an essential initiator of tumor angiogenesis [10,11]. Hypoxia leads to the increased expression of angiogenic molecules in hypoxic cells, including growth factors, cytokines, bioactive lipids, and matrix-degrading enzymes, which bind to receptors on adjacent vascular endothelial cells (ECs) to initiate new blood vessel formation [9,12].

Hypoxia-inducible factor (HIF) is highly expressed in the tumor microenvironment. High expression of HIF induces the up-regulation of angiogenic factors, such as vascular endothelial growth factor (VEGF), angiopoietin (Ang1 and Ang2), VEGF receptor (VEGFR), and Tie2 receptor at the transcriptional level, thereby, promoting the formation of new blood vessels in cancer [13,14]. After tumor neovascularization, adequate vasculature and blood supply continue to provide oxygen and nutrients to cancer cells, leading to tumor growth, progression, and metastasis [2,13].

Thus, inhibiting tumor angiogenesis can reduce the blood flow required for tumor development, and tumor cell growth will cease due to the lack of nutrients and growth factors needed to support the formation of newly formed blood vessels [15]. Previous studies have identified a number of angiogenic factors. The most widely studied modulators of angiogenesis are VEGF and the corresponding membrane receptors, primarily VEGFR2. These play a major role in regulating physiological and pathological angiogenesis [16]. The first treatment that targeted tumor angiogenesis was monoclonal antibody bevacizumab, which acts by interacting and blocking VEGF interaction with its receptor [17,18].

Another strategy to target VEGFR2 is to use small molecules, such as tyrosine kinase inhibitors (TKIs). This strategy resulted in the first clinically approved small-molecule-like drugs targeting tumor angiogenesis: sunitinib and sorafenib [19,20]. However, the high expectations for these anticancer drugs were quickly shattered, and treatment resistance complicates the use of VEGF signaling pathway inhibitors. They have shown only marginal benefits in early clinical trials, as escape or major resistance mechanisms are acquired, resulting in transient therapeutic benefits [18].

For example, sunitinib is widely used as a first-line treatment for metastatic renal cell carcinoma. However, 20–30% of patients do not respond to sunitinib, and even those who initially respond to sunitinib frequently experience disease exacerbations within a year [21]. Treatment failure can be attributed to adaptive mechanisms of the tumor microenvironment (e.g., the induction of compensatory angiogenic pathways) [22]. In addition, the tumor endothelium is a heterogeneous cell population with distinct functional and organ-specific phenotypes, indicating multiple pathological features of tumor vasculature [23]. In addition to vascular sprouting, other angiogenic processes, such as vessel co-option or vasculogenic mimicry, may play an important role in antiangiogenic therapy resistance [22].

The missing signaling molecules can be replaced in tumors by activating alternative pathways. For example, when VEGF/VEGFR is inhibited, angiogenesis can be maintained by secreting alternative factors, such as platelet-derived growth factor (PDGF) [24], basic fibroblast growth factor (bFGF) [25], and Ang2 [26], or by recruiting pro-angiogenic cells, such as trypsin-secreting mast cells, and promoting tumor growth [27]. This will reduce the effectiveness of single-target therapy or be ineffective. Recently, the Ang–Tie signaling pathway emerged as an attractive vascular drug target.

The Ang–Tie pathway is required for lymphatic and vascular development in mid-gestation and controls vascular permeability, inflammation and pathological angiogenic responses in adult tissues [28,29,30]. Drugs targeting the Ang–Tie pathway could complement current antiangiogenic strategies in the treatment of cancer [31]. In addition, the Ang–Tie pathway has a unique role in controlling vascular stability.

Thus, modulation of this axis may be beneficial in conditions where vascular overgrowth is not a problem but vascular stabilization is critical. In addition, studies using dual- or multi-targeted antibodies that simultaneously inhibit several angiogenic signals have shown incremental antiangiogenic efficacy in different tumor types [32,33,34,35]. However, many processes and factors that contribute to ineffectiveness and resistance to angiogenesis inhibitors remain, particularly those associated with the tumor endothelium.

We are faced with a multifaceted network of treatments in antiangiogenic therapy, many of which still need to be thoroughly investigated. At present, the combination of anti-tumor angiogenesis drugs and other therapies has become an important means in cancer treatment. Increased expression of heat shock proteins (HSPs) is another important defense mechanism activated in response to hypoxia. HSP inhibitors have potential antiangiogenic effects in cancer therapy [36].

A variety of HSP90 inhibitors have been developed and have demonstrated convincing antitumor activity in preclinical tumor models. Results from recent studies suggested that HSP90 inhibitors induce antiangiogenic properties by affecting the PI-3K/Akt/eNOS signaling pathway in endothelial cells, as well as through the down-regulation of VEGFR-2 expression [37]. Blockade of HSP90 also reduces the secretion and expression of tumor-cell-derived pro-angiogenic growth factors and cytokines, resulting in an “indirect” antiangiogenic effect [37]. In addition, angiogenesis inhibitors combined with HSP inhibitors may enhance the overall antiangiogenic activity [38].

This article reviews the commonly used drugs targeting VEGF/VEGFR and Ang/Tie and the clinical studies targeting both targets simultaneously, as well as several novel dual-target anti-tumor angiogenesis drugs. Several HSP inhibitors are also introduced. On the one hand, problems related to the development of drug resistance may be solved. These studies provide an important reference for the treatment of tumors and are crucial for the best treatment outcomes of cancer patients.

## 2. Mechanisms of Tumor Angiogenesis

During the development of solid tumors, tumor cells proliferate rapidly and consume a large amount of nutrients. In addition, high oxygen consumption, lack of nutrients, and the accumulation of metabolites in cells can create a hypoxic microenvironment that is not suitable for tumor cell growth [39]. As oxygen is key to the growth of any cell, tumor cells that are deprived of oxygen do not proliferate and divide [2]. HIF transcription is known to be induced under hypoxia. First, hypoxia stabilizes HIF1α, promoting its heterodimerization with HIF1β, and thereby transcriptionally activating many genes [13,40].

A large number of genes are transcriptionally regulated through the HIF pathway, including genes for various physiological functions, such as cell survival, proliferation, metabolism, inflammatory cell recruitment, and angiogenesis [41,42]. In tumor therapy, genes related to angiogenesis are major downstream targets that have been extensively studied. Two important angiogenic factors induced by hypoxia include vascular endothelial growth factor-A (VEGFA) and angiopoietin-2 (Ang2) [14].

### 2.1. Hypoxia-Inducible Factor (HIF)

HIF is the main trigger for the growth of new blood vessels in malignant tumors, and hypoxia is the most common mechanism of HIF activation in tumors [43]. Hypoxia due to an imbalance between tumor cell oxygen consumption and supply is present in 50–60% of solid tumors [44]. HIF is a heterodimer composed of an oxygen-dependent alpha subunit (HIF-α) and an oxygen-dependent beta subunit (HIF-β). There are three isoforms of HIF-α: HIF-1α, HIF-2α, and HIF-3α [45]. There are two isoforms of HIF-β, also known as aryl hydrocarbon receptor nuclear translocators (ARNT), namely HIF-1β and HIF-2β [46]. The alpha subunit is oxygen-sensitive, while the beta subunit is ubiquitously expressed [47]. Among them, HIF-1α is mainly responsible for activating transcriptional responses under hypoxia and is the most important among these HIFs [48].

HIFs are proteins that sense and respond to hypoxia by acting as transcription factors. An early insight into the possible mechanism of oxygen sensing in HIF was the determination that HIFα subunit stability is largely regulated by a 200 amino acid region known as the oxygen-dependent degradation domain (ODD) [49]. Conserved proline residues on the α subunit are hydroxylated by prolyl-4-hydroxylase (PHDs) in the presence of oxygen, thereby, promoting HIF-α degradation [50,51]. α subunit hydroxylation as a recognition signal for the von Hippel-Lindau (pVHL) tumor suppressor protein, which subsequently targets the α subunit for degradation by 26S proteasome [52,53].

However, under hypoxic conditions, PHD was unable to hydroxylate the α subunit. This results in HIF-α protein stabilization, nuclear translocation, and dimerization with HIF-1β to form the HIF transcription factor [40]. In the nucleus, HIFs bind to the A/GCGTG consensus motif in the promoter region of target genes, and these sites are called hypoxia response elements (HREs). By recruiting transcriptional coactivators, HIFs regulate the expression of numerous genes involved in diverse processes, including angiogenesis, metabolism, erythropoiesis, apoptosis, pH regulation, metastasis, and cell differentiation [54,55]. HIF transcriptional activity is also regulated by a second oxygen-sensitive hydroxylation event mediated by factor inhibiting HIF-1 (FIH-1) [56,57].

FIH-1 is a 2-oxoglutarate-dependent oxygenase (similar to PHD) that catalyzes the hydroxylation of asparagine residues in the C-TAD of HIF-α, preventing interaction with coactivators [58]. Due to specific amino acid differences between the two HIFs, HIF-1α is more sensitive to FIH-1-mediated inhibition than HIF-2α is [59]. Hypoxia-associated factor (HAF) is an E3 ubiquitin ligase that switches HIF-1-dependent signaling to HIF-2 by targeting HIF-1α degradation and increasing HIF-2α transactivation [60].

Although the primary mode of HIF stabilization is through proline hydroxylation and VHL-mediated degradation, several non-hypoxia-driven stimuli, such as growth factors, cytokines, hormones, and various stressors also regulate HIF [47]. For example, several growth factors and their cognate receptors, conducted through the PI3K or Ras/MAPK pathways, can induce HIF expression [61,62]. HIFs are direct substrates of multiple protein kinases that regulate HIF stabilization, nuclear translocation, and activation [63]. In addition, studies have also shown that ROS can directly regulate the expression of HIFs [64].

HIF-1α plays an important role in endothelial cell (EC) biology and angiogenesis. The deletion of HIF-1α prevents EC angiogenic behavior, including proliferation, migration, chemotaxis, and wound healing [48]. The angiogenesis of many solid tumors is related to HIF-1α [65,66,67]. In addition, HIF-2α has also been shown to be involved in tumor angiogenesis. HIF-2α deficiency reduces vascular function and tumor angiogenesis in mice EC [68]. VEGF expression is positively regulated by HIF-2α in VHL-deficient RCC cells [69]. HIF-2 promotes hypoxia-mediated angiogenesis and breast cancer metastasis through the induction of long noncoding RNA in breast cancer [70]. Collectively, these findings suggest that both HIF-1α and HIF-2α contribute to tumor angiogenesis.

HIF induces the expression of numerous pro-angiogenic factors, including VEGF, Ang1/2, VEGFR (FLT-1 and FLK-1), Tie2 receptors, PDGF-B, plasminogen activation inhibitor-1 (PAI-1), and matrix metalloproteinases (MMP-2 and MMP-9). [71]. The HIF-1α pathway has been shown to be a master regulator of vasculature formation by upregulating these factors, such as VEGF [72].

### 2.2. VEGF and Ang

While vascular growth and maturation are complex and highly coordinated processes requiring sequential activation of multiple factors, it is agreed that VEGF and Ang signaling are critical steps in tumor angiogenesis [43]. HIF-1α can transcriptionally activate several pro-angiogenic molecules by directly binding to their promoters.

#### 2.2.1. VEGF/VEGFR

Of all these pro-angiogenic factors activated by HIF, VEGFA, a potent endothelial mitogen, is the most prominent protein because it is highly expressed in many human tumors [73,74]. In tumors, such as neuroblastoma, HIF-1 mediates acute hypoxia-induced VEGF expression, whereas HIF-2 regulates VEGF expression during chronic hypoxia [75]. “VEGF” refers to both the originally identified disaccharide protein (now known as VEGFA) and the family of VEGF-related polypeptides [76]. VEGFA is produced by most cells in the body but is upregulated in hypoxia. In tumors, VEGF is produced by hypoxic tumor cells, ECs, and infiltrating myeloid cells called tumor-associated macrophages (TAMs). In mammals, the VEGF family has five members: VEGFA, VEGFB, VEGFC, VEGFD, and placental growth factor (PGF) [77].

VEGF family members bind to the three receptor tyrosine kinases (VEGFR1, VEGFR2, and VEGFR3) in an overlapping manner. VEGFR1 and VEGFR2 are mainly expressed on vascular ECs; however, VEGFR3 is especially expressed on lymphatic ECs. VEGFR2 has stronger proangiogenic activity and higher tyrosine kinase activity than does VEGFR1 [78]. VEGFA mediates in vivo angiogenic responses primarily through the activation of VEGFR2 [76]. VEGF receptors can be divided into three domains: the extracellular VEGF-binding domain, transmembrane domain, and intracellular domain (tyrosine-activation domain) [79].

The expression of VEGFA, like all other genes, is regulated in terms of transcription, mRNA stability, and translation. Transcriptional regulation of the VEGF gene is mediated through the proximal region of the promoter (−88 bp upstream of transcription initiation), which contains a high proportion of the GC domain. These regions can bind to specific protein (Sp) family factors and AP-2 transcription factors [80].The transcription of VEGF is by recruiting HIF to the promoter of VEGF through the phosphorylation of transcription factor Sp1 and the HIF-1α subunit [80,81].

Transcription factor activator protein-1 (AP-1) can also alter the transcription of VEGF [80]. VEGFA mRNA is stabilized by the stress-activated kinase p38. Translation is ensured by an internal ribosome entry site (IRES) sequence under hypoxic conditions [82]. HIF-2 also forms heterodimers with the aryl hydrocarbon receptor nuclear translocator and regulates VEGF expression [83]. In addition, the expression of VEGFR2 also promotes angiogenesis under hypoxic conditions. Hypoxia does not increase the expression of VEGFR2 through HIF-induced transcription but increases phosphocortin-like 3 (PDCL3) production to stabilize VEGFR2 protein expression [84].

VEGF is mainly produced by cells that surround blood vessels, and it acts on endothelial cells through a paracrine mechanism [85]. After VEGF-A levels reach maximal concentration levels at the leading edge of the vascular sprout, it binds to VEGFR2 and induces the migration of endothelial tip cells. Once VEGFR is activated, it leads to a series of downstream pathways related to tumor angiogenesis, including endothelial cell proliferation; survival, invasion, and metastasis; cytoskeletal rearrangement; and vascular permeability [6,86].

The transduction of downstream Raf-MEK-MAPK, P13K/AKT, and ERK1/2/FAK signals affects endothelial cell proliferation and survival [87,88,89]. The migration of endothelial cells is an important prerequisite for tumor angiogenesis. PI3K/Akt signaling is responsible for the expression of other molecules required for tumor cell invasion and metastasis, including Cdc42, Rho, and Rac proteins [76,90,91].

VEGFA can activate c-Src and Yes proteins through VEGFR and phosphorylated adhesion factors, such as VE-cadherin and β-catenin, in the presence of TSAd to increase vascular permeability [92]. Furthermore, activated endothelial nitric oxide synthase (eNOS) affects the vascular permeability by releasing nitric oxide in blood vessels. VEGF can also activate PLCγ through PI3K/Akt, thereby, activating the nuclear factor of T cells to regulate the intracellular calcium concentration or increase eNOS production to increase vascular permeability [93,94]. Enhanced vascular permeability will maintain an adequate nutrient and oxygen supply, thereby, resulting in rapid tumor growth [95].

**Figure 1:**
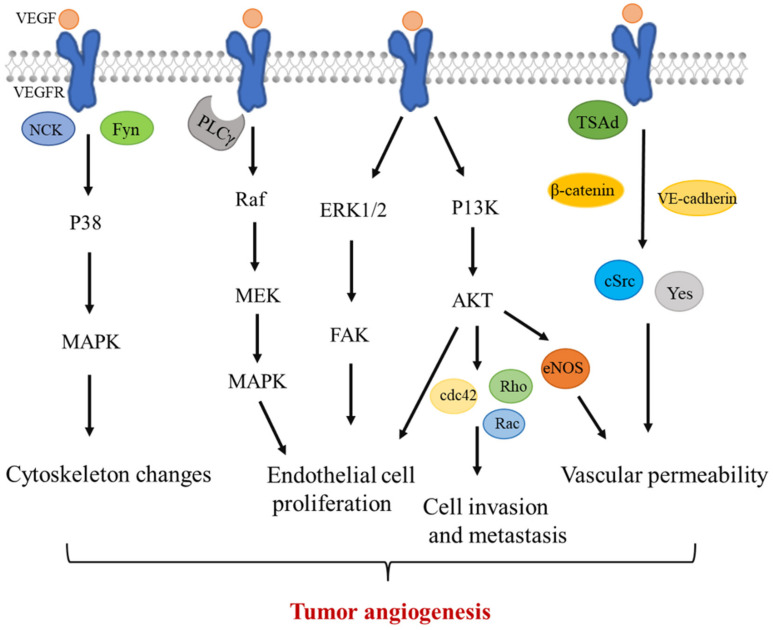
VEGF signaling pathway. After the VEGF level reaches the maximal concentration level at the leading edge of the vascular sprout, it binds to VEGFR and induces the migration of endothelial tip cells. Once VEGFR is activated, it leads to a series of downstream pathways. The transduction of downstream Raf-MEK-MAPK, P13K/AKT, (ERK)1/2/FAK, and other signals affects endothelial cell proliferation and survival. PI3K/Akt signaling is responsible for the expression of other molecules required for tumor cell invasion and metastasis, including Cdc42, Rho, and Rac proteins. VEGFA can activate c-Src and Yes proteins through VEGFR and phosphorylated adhesion factors, such as VE-cadherin and β-catenin, in the presence of TSAd to increase the vascular permeability. Furthermore, activated endothelial nitric oxide synthase (eNOS) affects vascular permeability by releasing nitric oxide in blood vessels. VEGFR can activate the P38/MAPK signaling pathway through Nck and Fyn binding, induce changes in the cytoskeleton, and promote tube formation in endothelial cells. VEGF, vascular endothelial growth factor; and VEGFR, vascular endothelial growth factor receptor.

VEGFR can also activate the P38/MAPK signaling pathway through Nck and Fyn binding, induce changes in the cytoskeleton, and promote tube formation in endothelial cells [96] (Figure 1).

Experiments with VEGF deletion in mice and VEGF knockouts in zebrafish demonstrated the role of the VEGF pathway in the development of the blood and lymphatic vasculature. The genetic deletion of VEGFA or its major signaling receptor VEGFR2 results in early embryonic lethality (around embryonic stage E9) associated with a near-complete blockade of hematopoiesis and vascular development [97]. VEGF plays an important role in the female reproductive cycle.

The study found that melatonin (Mel) and follicle-stimulating hormone (FSH) act on secondary follicles and antral follicles, respectively, to promote follicular angiogenesis by increasing the expression of VEGF [98]. However, abnormal angiogenesis often has an important relationship with VEGF, which plays a key role in tumor angiogenesis. Research on breast cancer found that VEGF expression occurs throughout the tumor stage [99]. Most non-small cell lung cancer (NSCLC) cells overexpress VEGFA, and brain-derived neurotrophic factor (BDNF) enhances VEGF-dependent angiogenesis [100].

#### 2.2.2. Ang/Tie2

Hypoxia also upregulates Tie2 expression in human tumors [101]. The receptor tyrosine kinases (RTKs) Tie1 and Tie2 were discovered by screening endothelial cells (ECs) for expressed tyrosine kinases [102,103]. Gene targeting studies show that the Ang–Tie2 system plays a critical role during vascular remodeling and maturation and stabilization of the cardiovascular system [104,105]. Angiopoietins are a family of growth factors that regulate tumor angiogenesis through Tie2 receptors and are highly expressed in growing blood Ecs [28,106]. The Ang family includes four proteins: Ang1, Ang2, Ang3, and Ang4. However, the function of Ang4 (or its mouse homolog Ang3) has not been extensively studied [28].

Ang1, Ang2, Tie1, and Tie2 are required for vascular remodeling and maturation during development. Ang1 and Tie2 are required for cardiac development, and Ang2 and Tie1 are also required for lymphoid development after VEGF-directed signaling to form the primary vascular plexus [107,108]. Ang1 and Ang2 have completely different mechanisms of action. Among them, Ang2 is more likely to lead to tumor angiogenesis. Ang2 is induced by HIF under hypoxic conditions and acts as an antagonist ligand for Tie2 in endothelial cells [109]. Ang2 mediates the capillary destabilization required to initiate sprouting angiogenesis, blocks Tie-2 signaling, and allows VEGFA-induced cell migration and division [82,109].

**Figure 2:**
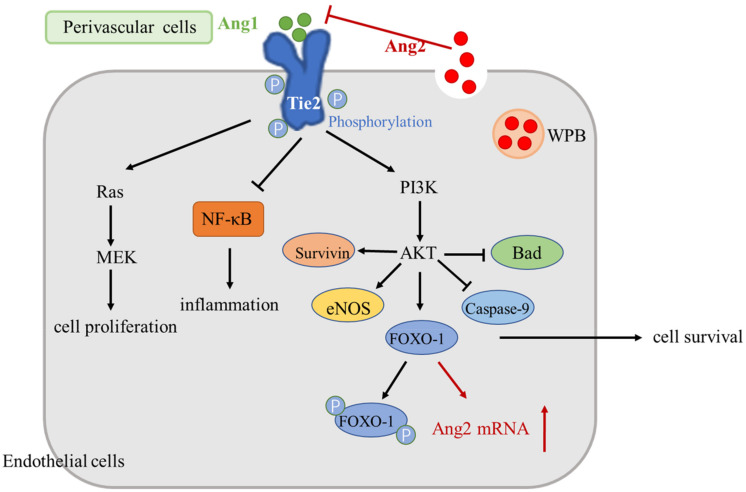
Angiopoietin signaling pathway. When Ang1 binds to Tie2, Tie2 auto-phosphorylates. Tie2 receptors regulate downstream signaling pathways, such as PI3K/AKT, MAPK)/ERK (also known as Ras/Raf/MEK/ERK), Survivin, and eNOS, and inhibits Caspase-9 and Bad, among others. These pathways are involved in reducing angiogenesis and vascular permeability, favoring vascular stability. Following Tie2 activation, FOXO-1 is phosphorylated and inactivated, thereby, promoting endothelial cell quiescence, survival, and vascular stability. In addition, the phosphorylation of Tie2 also prevents NF-κB signaling activation. In activated endothelial cells, Ang2 is released from endothelial Weibel–Palade bodies. It will antagonize Ang1–Tie2 signaling and inhibit Tie-2 phosphorylation, leading to vascular instability, vascular leakage, inflammation, etc., thereby promoting angiogenesis. Under these conditions, the FOXO-1 transcription factor is activated and promotes the transcription of Ang2 mRNA, further promoting vascular destabilization. Ang, Angiopoietin; and WPB, Weibel–Palade bodies.

In vivo, constitutive Angl–Tie2 signaling is thought to limit angiogenesis in mature blood vessels. When Ang1 binds to Tie2, five tyrosine residues within the intracellular kinase domain of Tie2 become auto-phosphorylated. The activation of Tie2 stimulates several signaling pathways, such as PI3K/AKT, mitogen-activated protein kinase (MAPK)/ERK (also known as Ras/Raf/MEK/ERK), survivin, eNOS, caspase-9, and Bad. [110,111]. These pathways are involved in reducing angiogenesis and vascular permeability, which are beneficial to vascular stability. Following Tie2 activation, FOXO-1 is phosphorylated and inactivated, thereby, promoting EC quiescence, survival and vascular stabilization [112]. In addition, phosphorylation of Tie2 also prevents NF-κB signaling activation [102] (Figure 2).

However, once ECs are activated, Ang2 is released from endothelial Weibel–Palade bodies (WPB). This may antagonize Ang1 and promote EC responses to exogenous cytokines, such as VEGF or tumor necrosis factor (TNF)-α [113,114,115,116]. Ang2 competes with Ang1 for binding to Tie2, inhibits Tie-2 phosphorylation, and adversely affects the PI3K/AKT and other pathways mentioned in the previous paragraph, leading to vascular instability, vascular leakage, and inflammation and thereby promoting angiogenesis.

It also can promote the development and metastasis of tumors by inducing the production of endothelial cell proteases and MMP-2 [117,118]. Under these conditions, the FOXO-1 transcription factor is activated and promotes the transcription of Ang2 mRNA, further promoting vascular destabilization [102]. In addition, the produced Ang2 protein will continue to be stored in the WBP, ready to be released into the extracellular matrix upon the detection of inflammatory signals [119].

Furthermore, Ang2 is expressed prior to VEGFA in growing tumor vessels and enhances angiogenesis in the presence of VEGFA [120]. Ang2 mediates the capillary destabilization required to initiate sprouting angiogenesis, thereby, blocking Tie2 signaling and allowing VEGFA-induced cell migration and division [82,109]. The Ang/Tie system plays a critical role in the pathophysiology of tumor vasculature as well as normal vasculature, and Ang2 expression was found to be upregulated in many types of cancer [121,122].

Compared with tumors in wild-type mice, Ang2-knockout mice exhibited a more mature phenotype with increased numbers of PCs and narrowed vessels [123]. Studies have shown that Ang2 can also enhance tumor angiogenesis, promoting the expression of several growth factors, including VEGFA [28]. Some studies have shown that Ang2 cooperates with VEGFA to promote tumor angiogenesis and metastasis [28,106,124].

### 2.3. HSP90

Another important defense mechanism activated in response to hypoxia is the increased expression of HSPs. Elevated levels of HSP have been detected in many solid tumors, including non-small cell lung cancer, esophageal cancer, and lymphoma. [125,126,127]. HSPs are highly conserved molecular chaperone proteins originally thought to be stress-responsive proteins required for the survival of cells or organisms after exposure to heat stress [128]. HSPs provide transient protection from stress as chaperones by regulating protein folding to ensure the correct conformation and translocation [129].

Cellular stress, including hypoxia and oxidative damage, can activate HSPs [129]. At present, a large number of studies have found that HSP90 plays an important role in tumor angiogenesis, and HSP90 inhibitors are also used to treat tumors clinically. The P13K/AKT pathway is essential for promoting endothelial cell survival, and HSP90 plays an important role in the P13K/AKT angiogenesis signaling pathway [130].

A recent study showed that HSP90 is involved in VEGF-mediated signaling by interacting with eNOS and subsequently releasing NO from endothelial cells [131]. HSP90 is involved in the activation of AKT and eNOS and further defined as a scaffolding role for HSP90 in the formation of the AKT/PDK1/eNOS complex [37]. In addition, blocking HSP90 also led to the degradation of AKT, c-Raf-1, and ERK protein kinases, which are important components of angiogenic signaling [37].

The VEGF/VEGFR system appears to be a direct target of HSP90 inhibitors [132,133]. Pharmacological inhibition of HSP90 stimulates VEGFR2 degradation in primary endothelial cells and blocks VEGF-A-stimulated intracellular signaling through VEGFR2 [134]. It was mentioned above that HSP90 is involved in the activation of AKT and eNOS. Studies have found that the binding of HSP90 to eNOS and the activation of the PI3K/AKT pathway promotes Ang-1-induced phosphorylation of eNOS, production of NO, and eventual angiogenesis [135].

## 3. Drugs for the Treatment of Tumor Angiogenesis

At present, there are many drugs and methods for antiangiogenesis, and here we discuss several new drugs and clinical programs (Table 1).

### 3.1. Drugs Targeting VEGF/VEGFR

VEGF/VEGFR has been the main target of antiangiogenic drugs in cancer therapy. At present, many anti-tumor angiogenesis drugs targeting VEGF/VEGFR targets have appeared in the clinic, including bevacizumab, axitinib, sorafenib, sunitinib, and afebecept, for the treatment of various cancer types [173,174].

#### 3.1.1. Bevacizumab

Bevacizumab was the first antiangiogenic drug approved for clinical use. It is a humanized monoclonal antibody that targets all VEGFA isoforms to prevent angiogenic processes within tumors [6]. It has been shown to have therapeutic efficacy in a variety of malignancies, including colorectal cancer, glioblastoma, non-squamous small-cell lung cancer, cervical cancer, ovarian cancer, and metastatic breast cancer [6,136,137]. In addition, post-treatment outcomes have been shown to lead to relapse due to invasion and drug resistance, necessitating the need for combination therapy to consolidate and strengthen [137].

#### 3.1.2. Axitinib

Axitinib is a selective inhibitor of VEGFR1, VEGFR2, and VEGFR3 and a weaker inhibitor of platelet-derived growth factor receptor (PDGFR) and KIT [138]. In preclinical studies, axitinib showed inhibitory activity against VEGF-mediated endothelial cell survival and demonstrated potent antitumor activity against tumors [139]. However, the activity of axitinib is limited to a small number of tumors, including renal cell carcinoma and radioactive iodine-refractory thyroid cancer [138].

#### 3.1.3. Sorafenib

Sorafenib inhibits tumor growth and angiogenesis by targeting the RAF/MEK/ERK pathway and receptor tyrosine kinases. For the treatment of unresectable hepatocellular carcinoma and advanced renal cell carcinoma [140]. Sorafenib has been an effective first-line therapy in advanced hepatocellular carcinoma [141]. Adverse events identified in patients given sorafenib mainly included gastrointestinal, physical, and skin disorders. In severe cases, sorafenib can cause high blood pressure and abdominal pain [142].

#### 3.1.4. Sunitinib

Sunitinib is a small-molecule multiple tyrosine kinase inhibitor targeting VEGFR, PDGFR, stem cell factor receptor, colony stimulating factor 1 receptor, FMS-like tyrosine kinase receptor, and neurotrophic factor receptor [143,175]. The parallel inhibition of these receptors reduces tumor angiogenesis, thus, leading to cancer cell apoptosis. Currently, it has been used to treat renal cell carcinoma and gastrointestinal stromal tumors [144]. Sunitinib-related side effects are often associated with pulmonary toxicity, causing patients to have difficulty breathing and to cough [176].

#### 3.1.5. Aflibercept

Aflibercept is a soluble recombinant fusion protein consisting of the extracellular domains of VEGFR1 and VEGR2 fused to the Fc portion of human immunoglobulin G1 (IgG1) that neutralizes VEGFA, VEGFB, and PGF [177]. It was approved by the FDA in 2012 for the treatment of metastatic CRC. Aflibercept exhibits higher tumor suppressor activity compared with bevacizumab in patient-derived xenograft (PDX) models [145]. However, there are a number of limitations to treatment with VEGF inhibitors. First, VEGF inhibitors can produce side effects, including hypertension, atherosclerosis, bleeding, and proteinuria [6,137]. Second, VEGF inhibition leads to the upregulation of various other pro-angiogenic factors, such as PDGF, angiopoietin, and FGF, leading to the failure of antiangiogenic therapy [178]. For example, in mice treated with sunitinib, accelerated tumor metastasis and decreased overall survival were found [179]. Another study in glioblastoma multiforme found that bevacizumab treatment resulted in more aggressive tumor growth, possibly due to mechanisms such as the MET pathway activated by VEGF inhibition [180].

Third, VEGF inhibitors not only target tumor cells but also affect normal capillaries to a certain extent, causing their regression [181,182]. Fourth, vascular normalization may be short-lived [183] and may depend on the tumor and the dose of anti-VEGF drug used [184]. This may be because tumors evade anti-VEGF therapy through the upregulation of alternative angiogenic pathways, such as Ang2–Tie2 signaling. In addition, the amplification of pro-angiogenic genes, the secretion of various pro-angiogenic factors, and the recruitment of pro-angiogenic bone marrow-derived cells also contribute to the occurrence of antiangiogenic drug resistance of tumor cells [178]. Therefore, new antiangiogenic strategies must be developed to overcome the side effects and resistance of these drugs and improve the efficiency of treatment by targeting multiple cancer-related angiogenic mechanisms.

### 3.2. Drugs Targeting Ang/Tie2

At present, more safe and effective anti-tumor angiogenesis pathways and targets have been explored. Among many such targets, the Ang–Tie2 system is considered a successful surrogate for VEGF [28]. Ang1 and Ang2 are involved in angiogenesis, and Ang2 in particular may prove effective in treating tumors.

#### 3.2.1. Trebananib (AMG 386)

Trebananib (AMG 386) is a first-in-class peptide antibody that inhibits the interaction of angiopoietins 1 and 2 with their receptor Tie2. Trebananib consists of a biologically active peptide grafted onto the Fc region of IgG [185]. Improved progression-free survival (PFS) in combination with paclitaxel in patients with recurrent ovarian cancer, undoubtedly enhances the ability to treat this difficult-to-treat disease [147]. In addition, the treatment of fallopian tube cancer, peritoneal cancer, breast cancer, gastroesophageal cancer, and renal cell cancer have also developed to the clinical trial stage [148,186,187].

However, subsequent studies have found disappointing results from clinical studies targeting Ang1 and Ang2 using the bispecific peptibody trebananib. The results of the phase II study of adding trebananib to chemotherapy suggest that the vascular normalizing effect of an Ang1–Ang2 blockade is often insufficient to improve patient outcomes [149,186,187]. One possible reason for the clinical failure of a dual Ang1–Ang2 blockade is that inhibition of Ang1, the agonistic ligand of Tie2, may impair the vascular normalization benefit conferred by blocking the antagonistic Tie2 ligand Ang2 [188]. In addition, the study found that dual inhibition of Ang1 and Ang2 can lead to peripheral edema [189].

#### 3.2.2. CVX 060

CVX 060 is a monoclonal antibody that treats solid tumors by inhibiting Ang2 [150]. In preclinical studies, CVX 060 was evaluated for activity in the colon cancer cell line Colo-205, and tumor growth was significantly reduced by CVX 060 monotherapy [151]. Combination therapy of CVX 060 with VEGF inhibitors, such as axitinib or sunitinib, has made progress in metastatic renal cell carcinoma (RCC) [152].

#### 3.2.3. Nesvacumab (REGN 910)

Nesvacumab (REGN910) is a fully human IgG1 monoclonal antibody that specifically binds and inactivates the Tie2 receptor ligand Ang2 with high affinity but has not been shown to bind Ang1 [153]. REGN 910 was evaluated for its efficacy in solid tumors in a phase I study of 47 patients, none of which showed dose-limiting toxicity (DLT), and the drug showed an acceptable safety profile [153]. In tumor xenograft models, Nesvacumab significantly inhibited the growth of several tumor types, including PC3 (prostate), Colo205 (colorectal), and A431 (epidermoid carcinoma), in a dose-dependent manner [154].

### 3.3. Combination of Drugs Targeting VEGF/VEGFR and Ang/Tie2

Substantial evidence suggests that most tumors in human patients and experimental animal models have high expression and activation levels of VEGF-A/VEGFR2 and Ang/Tie2 and that these systems interact synergistically on tumor angiogenesis and metastasis [28,106,124,190]. Activation of the Ang2–Tie2 axis may serve as an escape mechanism for anti-VEGF therapy [191]. Research found that circulating levels of Ang2 transiently decreased during the normalization window, recovered, or were even upregulated in mouse glioma tumor cells with progression following anti-VEGF treatment [192].

Furthermore, Ang2 overexpression in mouse glioma tumor cells inhibited the beneficial effects of anti-VEGFR2 therapy on tumor vessel normalization, brain edema, and animal survival by increasing vascular permeability [191]. Inhibition of Ang2 reduces tumor vascular sprouting, whereas anti-VEGF antibody induces vascular regression [193]. The effects of blocking Ang2 and VEGF appear to be at least partially complementary. Furthermore, the combination of Ang2 inhibition with VEGF drugs showed significantly enhanced antitumor effects compared to monotherapy [194,195]. Currently, multiple clinical trials are underway to investigate the dual inhibitory effects of VEGF and ANG2 in cancer patients.

#### 3.3.1. Nesvacumab (REGN910) plus Aflibercept

Nesvacumab is a selective human Ang2 MAb that potently blocks Ang2 signaling through the Tie2 receptor. Aflibercept is a recombinant human fusion protein and a decoy receptor for VEGFA, VEGFB, and PGF. In a mouse xenograft model, the combination significantly inhibited tumor growth and angiogenesis compared to either drug alone. Proteinuria was found to be dose-related; however, this symptom subsided with dose adjustment. The coadministration of two drugs is generally well tolerated in patients with advanced cancer [196].

In Colo205 and MMT models, the combination of nesvacumab and afibercept (VEGF Trap) was also found to significantly outperform the single agent in inhibiting tumor growth and promoted significant regression of Colo205 tumors. Consistent with this combination’s potent effect on tumor growth, nesvacumab plus afibecept also reduced Colo205 tumor vascularity and tumor perfusion more significantly than the single agent. These results suggest that nesvacumab is a promising candidate for selectively inhibiting tumor angiogenesis, either as a single agent or in combination with anti-VEGF therapy, even in tumor models (Colo205) that are very sensitive to anti-VEGF therapy [154].

#### 3.3.2. Trebananib plus Bevacizumab

Although clinical studies targeting both Ang1 and Ang2 with trebananib alone are not entirely satisfactory. However, tumor xenograft studies with trebananib showed that dual inhibition of Ang1 and Ang2 in the presence of a concurrent VEGF blockade was significantly more effective than inhibition of either target alone [148,197,198]. In a phase I/II study in glioblastoma, the PFS at 6 months after completion of the trebananib and bevacizumab combination was 24% compared to 0% with trebananib alone [199] and was 23% PFS after combination therapy in a phase II study in adult glioblastoma, gliosarcoma, and oligodendroglioma [199]. However, in a phase 2 study of HER2-negative locally recurrent or metastatic breast cancer, the addition of trebananib to paclitaxel and bevacizumab did not significantly prolong the estimated PFS [148].

#### 3.3.3. Trebananib plus Sorafenib

Interim results from a phase 1b study showed an acceptable toxicity profile and possible antitumor activity in patients with metastatic renal cell carcinoma treated with sorafenib or sunitinib plus trebananib [200]. The tolerability and antitumor activity of trebananib plus sorafenib in previously untreated patients with clear-cell metastatic renal cell carcinoma was evaluated in another phase 2 study. In these patients, trebananib plus sorafenib was tolerable but did not significantly improve the PFS compared with placebo plus sorafenib [187]. A phase II study in patients with advanced hepatocellular carcinoma also showed no significant improvement in PFS rates at 4 months with the two-drug combination compared with sorafenib alone [201].

### 3.4. Drugs Targeting Both VEGF/VEGFR and Ang/Tie2

Currently, dual-target inhibitors targeting both VEGFA/VEGFR2 and Ang/Tie2 have been studied and developed. In addition to the pharmacoeconomic advantages and convenience, bispecific-targeted drugs have other advantages over the therapeutic application of monospecific-targeted drugs or their combinations. Systemic toxicity can be reduced by targeting disease sites and modulating internalization properties.

Additionally, effector cells can be recruited, and synergistic effects are seen when targeting cell surface receptors [202]. The use of bispecific antibodies also facilitates targeting several pathways simultaneously to avoid escape and resistance mechanisms [203]. Therefore, here, we will focus on this type of drug. However, these dual-target inhibitors are only in the preclinical test stage, and no corresponding drugs have been developed. This will be the direction of scientists’ efforts in the future.

#### 3.4.1. CVX-241

CVX-241 is a first-in-class bispecific antibody developed in 2010. The CVX-241 diabody is produced by adding two short peptides that inhibit VEGF or ANG2 to a branching linker, which is then linked to the antibody. It utilizes a technology called bispecific CovX antibodies, a multifunctional technology based on scaffolded antibodies and pharmacophore peptide heterodimers that enables rapid generation and chemical optimization of bispecific antibodies [150]. To demonstrate the specificity of CVX-241, direct binding ELISA was used to examine the binding of CVX-241 to the VEGF family and the angiopoietin family.

CVX-241 selectively binds human VEGFA and Ang2 but not human VEGFB, VEGFC, VEGFD, Ang1, Ang4, or Ang3 [150]. Pharmacokinetic evaluations in mice, rats, and monkeys showed that CVX-241 confers an antibody-like half-life to the peptide in animals, opening up the possibility of once-weekly dosing in humans [150]. Bispecific CVX-241 inhibited VEGF-VEGFR2/Ang2–Tie2 interactions compared to monospecific CovX antibodies and further demonstrated efficacy in colon adenocarcinoma xenograft models [150,204].

In addition, the antitumor efficacy of CVX-241 was also evaluated in the MDA-MB-435 breast cancer xenograft model and the A431 skin cancer xenograft model [150]. However, during the phase Ⅰ clinical trial, it was terminated early due to lack of pharmacological effects. A study found that, in the LM2-4 breast cancer model, adjuvant CVX-241 to sunitinib improved the overall survival in mice [152]. At the same time, it also reminds us that CVX-241 can be used as an adjuvant to continue clinical experimental research in the future.

#### 3.4.2. Vanucizumab

Vanucizumab (RO5520985, RG7221), a novel bevacizumab-based bispecific human IgG1 antibody, acts as a dual-targeted inhibitor of two key angiogenic factors, VEGF-A and Ang-2 [205]. First studied in 2011, through a new method of producing heterobivalent bispecific human IgG1 antibodies (CrossMabs), bispecific antibodies with minimal differences from natural antibodies were developed [203]. The antibody displays a classical IgG structure and exhibits favorable IgG-like properties in terms of pharmacokinetics, diffusion, tumor penetration, production, and stability [203]. In 2012, vanucizumab became one of the first human heterodimeric bispecific IgG antibodies to enter clinical trials in cancer patients [206].

Studies in mouse models of Colo205 tumors (an established model of anti-Ang-2 therapy) and advanced KPL-4 tumors found that vanucizumab treatment reduced the tumor vascular density, stabilized vascular architecture, eliminated hypoxia, and reduced the amount of metastatic spread of leaky vessels [205]. Evaluating the safety of vanucizumab, Ang-2-VEGF-A inhibition does not exacerbate the adverse effects of anti-VEGF-A therapy on healthy blood vessels [205]. In addition, Ang-2-VEGF-A dual targeting was found to have better therapeutic effects on larger tumors compared with monotherapy [205]. This may be related to the fact that larger tumors contain different blood vessel types.

Different types of blood vessels have different degrees of sensitivity to anti-VEGFA treatment [207]. In athymic nude mice bearing subcutaneous rectal cancer xenograft tumors, the efficacy of vanucizumab co-targeting VEGF and Ang-2 in combination with chemotherapy in a chemoresistant colorectal cancer model was evaluated. The vanucizumab-containing regimen demonstrated clear advantages over clinical standard anti-VEGF/chemotherapy regimens [208]. Furthermore, the potential for direct equimolar and pharmacoeconomic dosing regimens achieved by vanucizumab was compared to the combination of monospecific antibodies [205].

In 2018, the first human phase I study was conducted in adult patients with advanced solid tumors [155]. Acceptable safety and tolerability was found in advanced adult cancer patients consistent with bevacizumab and Ang/Tie2 axis-selective inhibitors [155]. In preclinical studies, vanucizumab was shown to have significant antitumor, antiangiogenic, and antimetastatic effects [205]. In a clinical analysis of patients with advanced solid tumors, such as colorectal cancer, non-squamous non-small cell lung cancer, breast cancer, gastric cancer, pancreatic cancer, and melanoma, treated with vanucizumab, soluble markers of angiogenesis were affected by vanucizumab administration, infusion post-reduction in circulating levels of free (unbound bioactive) VEGF-A.

In addition, Ang-2 confirmed the mechanism of action of vanucizumab and illustrates the potent effect of vanucizumab against tumor vasculature [156]. However, a study of metastatic colorectal cancer found that, compared with bevacizumab, neither PFS nor the overall response rate (ORR) improved with vanucizumab in patients treated with modified (m) FOLFOX-6 folinic acid (leucovorin) combination therapy [192]. The failure to meet the primary endpoint of this clinical trial may have been due to the use of high doses of vanucizumab.

Some previous studies have found that the dose of anti-VEGF drugs is important and that high doses of anti-VEGF drugs can lead to the increased deposition of extracellular matrix as well as to immunosuppression [209,210,211]. In contrast, the use of lower doses of antiangiogenic agents (e.g., as low as one-quarter of the doses that induced antiangiogenic effects in animal studies) has the potential to induce the long-term normalization of blood vessels [183,212]. Thus, using lower doses of vanucizumab may provide better treatment over a long period of time. The efficacy and safety profile of vanucizumab suggest that it is a promising antitumor, antiangiogenic, and antimetastatic agent. However, there are few studies on vanucizumab, and these are only preliminary at present. Further clinical studies are needed in the future.

#### 3.4.3. Faricimab

Faricimab (RO6867461, RG7716) is a VEGFA and Ang2 bispecific antibody, mainly used for the treatment of wet or neovascular age-related macular degeneration (AMD) and diabetic macular edema (DME) and other ocular diseases [157,158,213]. It has been accepted by the Food and Drug Administration (FDA) and approved for marketing in January this year. The safety and efficacy of faricimab were compared to ranibizumab in a study in patients with treated macular edema. This study concludes that the dual inhibition of angiogenic factors proved more effective than single inhibition of factors in macular edema [158]. Faricimab was evaluated in a phase II study in patients with age-related macular edema. A total of 65% of patients treated with fariximab showed no symptoms of the disease, and no new or unexpected toxicities were observed [159]. However, regarding whether it helps to relieve tumor angiogenesis and thus has an anti-tumor effect, there is no relevant research. This requires further research.

#### 3.4.4. BI836880

BI836880 is a novel humanized bifunctional nanobody (engineered antibody fragment of variable antibody domain) developed by Boehringer Ingelheim, whose domain can bind to VEGF and Ang-2 in a manner similar to Faricimab, and has an Albumin-binding domain that prolongs the half-life and shows preclinical activity in cancer models [160]. The first human Phase I clinical trial was conducted in 2018, and early signs of antitumor activity were observed [160]. Later, BI836880 combined with PD-1 inhibitor Ezabenlimab (BI754091) was explored for the treatment of advanced/metastatic solid tumors. The combination has a manageable safety profile, and preliminary anti-tumor activity was observed [161].

Brain metastases (BM) are a growing challenge in oncology, and nanobody BI836880 extended animal survival and reduced BM formation; however, extracranial metastases were not reduced [162]. The study also supports the idea that antiangiogenic compounds may be primarily effective in the brain because BM, especially in lung adenocarcinoma, shows a particularly stronger angiogenic response at this site [162,214,215]. BI836880 is predicted to make good progress in tumor therapy.

#### 3.4.5. Double Antiangiogenic Protein (DAAP)

Dual antiangiogenesis protein (DAAP) is a chimeric decoy receptor that binds to both VEGFA and angiopoietin and blocks their actions [163]. DAAP is a highly potent molecule for reversing tumor angiogenesis and metastasis in implanted and spontaneous solid tumors. Compared to VEGF-Trap or Tie2-Fc, which block VEGFA or Angiopoietin alone, DAAP appears to be well distributed in the tumor environment and blocks VEGFA and Ang2 in a synergistic manner [163] with more anti-tumor growth, antiangiogenic, and anti-metastatic effects in colon cancer and spontaneous breast tumor models.

In addition, it has a longer half-life than VEGF-Trap and Tie2-Fc and may be more cost-effective than dual anticancer agents [163]. It is also effective in reducing ascites formation and vascular leakage in ovarian cancer models [163]. Angiogenesis plays a key role in synovial inflammation and joint destruction in rheumatoid arthritis (RA). DAAP was also found to be effective in preventing inflammation and bone destruction and was therapeutically effective in mice alone or in combination with TNF-alpha inhibitors [164]. However, further preclinical studies are still lacking.

## 4. Combinations of HSP90 Inhibitors with Other Antiangiogenic Drugs

Historically, the maximum tolerated dose (MTD) has been used for the clinical development of drugs targeting the VEGF pathway [212,216] with high-dose, prolonged anti-VEGF therapy associated with lower levels of tumor perfusion and increased hypoxia [183]. Blocking the VEGF pathway by adding drugs targeting the ANG2–Tie2 pathway appears to prolong the window of vascular normalization [33,217].

Over time, however, tumors can become hypoxic again. Therefore, new methods need to be found to solve this problem. Many HSP90 inhibitors have been developed. Tanespimycin (17-AAG), a first-in-class HSP90 inhibitor, has entered Phase III clinical trials [218]. Tanespimycin inhibits the binding of HSP90 to HIF-1α and increases the binding of activated C kinase-1 (RACK1) receptor, which recruits elongin C and its E3 ligase complex to HIF-1α, resulting in ubiquitin–proteasome pathway degradation [219]. Tanespimycin can directly inhibit eNOS mRNA transcription in an in vitro human umbilical vein endothelial cell (HUVEC) model of angiogenesis; however, the mechanism remains unclear [220].

A clinical study found that tanespimycin causes tumor regression in patients with HER-2-positive metastatic breast cancer [165]. However, its hepatotoxicity, low solubility, and limited bioavailability make its use difficult in clinical practice [221]. Tanespimycin failed clinical studies in advanced prostate cancer and clear cell renal cell carcinoma [222,223]. The discordance between Hsp90-targeted efficacy in preclinical models and less favorable clinical outcomes may be due to a number of pharmacological factors. Tanespimycin is a substrate of the multidrug resistance (MDR) transporter P-glycoprotein and related multi-drug resistant associate protein (MRP) efflux pumps [224].

Cellular resistance acquired by this mechanism has been observed in cell cultures [166]. Many improved formulations and chemical derivatives have subsequently emerged based on Tanespimycin, including DMSO-free formulations, KOS-953, 17AAG (CNF-1010) an oil-in-water nanoemulsion, a reduced form of 17AAG (IPI-504), and transgenic derivatives, such as 17-dimethylaminoethylamino-17-demethoxygdamycin (17DMAG), etc. [165,167,225]. Several derivatives showed higher potency and lower toxicity compared to 17-AAG. Currently, there are no FDA-approved Hsp90 inhibitors on the market.

Subsequently, based on different chemical scaffolds, many novel synthetic HSP90 inhibitors have been developed. CNF2024 is a purine scaffold HSP90 inhibitor. It binds to the ATP-binding pocket of HSP90, leading to HSP90 chaperone dysfunction, which induces the degradation of client proteins and tumor growth inhibition [168]. A phase I dose-escalation study of orally administered CNF2024 was completed in patients with relapsed B-cell chronic lymphocytic leukemia (CLL), advanced solid tumors, or lymphoma [167].

SNX-5422 is a 6,7-indazol-4-one scaffold-based inhibitor [170]. SNX-5422 is a water-soluble, orally bioavailable prodrug of SNX-2112 that selectively binds to the ATP pocket [226]. SNX-2112 abrogates signaling through AKT and ERK, potently inhibits growth, angiogenesis and osteoclastogenesis in preclinical models of multiple myeloma and other hematological tumors [227]. In a Phase I study, the MTD and safety of SNX-5422 were evaluated in patients with advanced solid tumors [169].

AT-533 is a novel potent Hsp90 inhibitor. It can competitively bind to the ATP-binding pocket of Hsp90 and significantly inhibit Hsp90 activity, with higher solubility and pharmacological properties than 17-AAG [172]. The study found that AT-533 inhibited the viability in vitro and in vivo, tube formation, cell migration, invasion, and angiogenesis of HUVECs and was more potent than the Hsp90 inhibitor 17-AAG [171]. In studies conducted on breast cancer, AT-533 significantly inhibited the viability of breast cancer cells in vitro and the growth of breast cancer xenografts in vivo.

It suppressed the expression levels of HIF-1α and VEGF in breast cancer cells in vitro and in vivo, and induced the expression of apoptosis-related proteins in breast cancer xenografts. The findings suggest that the Hsp90 inhibitor AT-533 inhibits tumor angiogenesis by inhibiting breast cancer growth and blocking HIF-1α/VEGF/VEGFR-2-mediated signaling, thereby, triggering an antitumor response in breast cancer [171]. In addition, the study found that AT-533 attenuated herpes simplex virus (HSV)-1-induced inflammation and inhibited keratitis caused by HSV [172,228]. Studies have found that targeted therapy can lead to resistance to anti-VEGF drugs, while still remaining highly sensitive to HSP90 inhibition [38].

Furthermore, it simultaneously targets multiple proangiogenic regulators, potentially impairing tumor cell signaling [229]. Since HSP90 antagonists have potent antiangiogenic properties, the combined use of antiangiogenic drugs and HSP90 inhibitors may prove to be a valuable strategy for overcoming drug resistance [230]. However, this combined approach needs to be validated in further studies.

## 5. Conclusions

Angiogenesis plays an important role in tumor progression. The mechanism of angiogenesis is tightly regulated by highly specific angiogenic stimulators and inhibitors, as imbalances in the angiogenesis process can lead to severe pathological conditions. Common pro-angiogenic factors include VEGF, FGF, PDGF, Ang, and HIF. Activation of the signaling pathways of VEGF and ANG is considered to be a critical step in tumor angiogenesis. The effective inhibition of tumor angiogenesis may prevent tumor progression but may not eradicate tumors when single-mechanism antiangiogenic agents are used as stand-alone therapy. VEGF/VEGFR has been the main target of antiangiogenic drugs in cancer therapy.

Antineoplastic drugs, such as bevacizumab, axitinib, sorafenib, sunitinib, and afebeceptin, have been developed to treat various cancer types. However, these drugs have side effects and resistance. Scientists therefore need to overcome these by targeting multiple angiogenesis-related mechanisms. Ang/Tie-targeted inhibitors, including trebananib, CVX 060, and nesvacumab, are considered to be successful alternatives to VEGF. VEGF/VEGFR2 and Ang/Tie2 have demonstrated synergistic effects on tumor angiogenesis and metastasis. Numerous clinical studies have shown that the combination of Ang2 inhibition with VEGF drugs has enhanced antitumor effects.

At present, dual-target inhibitors targeting VEGFA/VEGFR2 and Ang/Tie2 have been researched and developed, including CVX-241, vanucizumab, faricimab, BI836880, and DAAP. These drugs have many advantages over the therapeutic application of monospecific targeted drugs or their combinations: (1) economic advantages and convenience, (2) less systemic toxicity, (3) synergistic effects are seen when targeting cell surface receptors, and (4) the simultaneous targeting of multiple pathways to avoid escape and resistance mechanisms. However, these dual-target inhibitors are still in preclinical or clinical trials.

HSP90 provides an attractive target for cancer therapy. Inhibition of HSP90 function results in the simultaneous disruption of many signaling pathways that are critical for both tumor progression and tumor angiogenesis. In addition, the VEGFR system and its downstream signaling molecules and survival factors are highly dependent on the function of HSP90. The addition of HSP90 inhibitors to conventional antiangiogenic therapy may be a valuable approach to overcome treatment resistance. However, continued preclinical and clinical studies are required.

## Figures and Tables

**Table 1:** Antiangiogenesis drugs.

Drug	Target	Disease/Model	Phases of Clinical Trials and Approval	References
Bevacizumab	VEGFA	renal cell carcinoma; colorectal cancer; Glioblastoma; non-small cell lung cancer	2004, approved	[136,137]
Axitinib	VEGFR1, 2, 3	renal cell carcinoma	2012, approved	[138,139]
Sorafenib	VEGF2, 3; PDGFR	hepatocellular carcinoma; renal cell carcinoma	2005, approved	[140,141,142]
Sunitinib	VEGFR1, 2, 3; PDGFR	renal cell carcinoma; gastrointestinal stromal tumor	2006, approved	[143,144]
Aflibercept	VEGFA, B; PLGF	colorectal cancer	2012, approved	[145,146]
Trebananib (AMG 386)	Ang2	fallopian tube cancer; breast cancer; gastroesophageal cancer; renal cell carcinoma	Phase II; completed	[147,148,149]
CVX 060	Ang2	glioblastoma	Phase II; withdrawn prior to enrolment	[150,151,152]
Nesvacumab (REGN 910)	Ang1, 2	advanced-stage solid tumors	Phase I, completed	[153,154]
CVX-241	VEGFA and Ang2	breast cancer xenograft model; skin cancer xenograft model; advanced stage solid tumors	Phase II; terminated owing to poor tolerability	[150,152]
Vanucizumab	VEGFA and Ang2	multiple orthotopic; subcutaneous xenograft models; Glioblastoma	Phase II; completed	[155,156]
Faricimab	VEGFA and Ang2	macular edema; macular degeneration	not used for cancer treatment	[157,158,159]
BI836880	VEGFA and Ang2	brain metastases	Phase I, completed	[160,161,162]
Double antiangiogenic protein (DAAP)	VEGFA and Ang2	colon cancer; spontaneous breast tumor models	preclinical stage	[163,164]
Tanespimycin (17-AAG)	HSP90	prostate cancer	Phase III; completed	[165,166]
CNF2024	HSP90	Hodgkin’s lymphoma	Phase I, completed	[167,168]
SNX-5422	HSP90	hematologic tumors	Phase I, completed	[169,170]
AT-533	HSP90	breast cancer	preclinical stage	[171,172]

Note: VEGF, vascular endothelial growth factor; VEGFR, vascular endothelial growth factor receptor; PDGF, platelet-derived growth factor receptor; Ang, angiopoietin; and HSP, heat shock protein.

## Data Availability

Not applicable.
